# Correlation between impostor phenomenon and burnout syndrome in medical residents: a single-center study

**DOI:** 10.1016/j.bjane.2025.844688

**Published:** 2025-10-15

**Authors:** Cristina González Ramírez, Rocío Pérez Bocanegra, Carlos Armando Sánchez-Navarro

**Affiliations:** Instituto Mexicano del Seguro Social, Hospital General de Zona 1, Anesthesiology Department, OOAD Aguascalientes, Mexico

Dear Editor,

Since the 1970s, two psychological constructs have been described: Impostor Phenomenon (IP), defined as the psychological experience of intellectual and professional fraudulence^1^ and Burnout Syndrome (BS), which is defined as the psychological syndrome that emerges in response to prolonged exposure to work stressors, characterized by three dimensions described as Emotional Exhaustion (EE), Depersonalization (DP) and decreased Personal Achievement (PA).^2^ For healthcare professionals, specifically physicians in training, the frequency of IP has been reported to be as much as 30%^3^ and the rate of BS from 25% to 75%. The failure to recognize and assess IP and BS can limit the career development process. Given the negative impact of IP and BS on healthcare professionals, we evaluated both constructs in a population of residents from all medical specialty programs, starting from the second year onwards, at the General Hospital of Zone 1 Aguascalientes of the Mexican Institute of Social Security, a second-level general hospital, to determine the frequency and correlation of IP and BS among the participants.

The study, approved by the Local Ethics and Research Committee (Registry Number R-2024-101-119), was an observational, descriptive, prospective, and single-center study. All participants provided duly informed consent.

A total of 56 second- and third-year medical residents from all medical specialty programs in the 2024–2025 academic year were selected. Demographic and academic data were obtained through a self-administered questionnaire, assessing six variables: age, gender, academic program, academic year, duty hours and rest hours. The Clance Impostor Phenomenon Scale (CIPS) was administered to all participants. The CIPS is a psychometrically validated instrument composed of 20 items rated on a 5-point Likert scale. CIPS Scores are categorized as follows: scores of 40 or below reflect a few characteristics of IP; scores between 41 and 60 indicate a moderate level; scores from 61 to 80 suggest frequent impostor-related experiences; and scores of 81 or above correspond to intense manifestations of the IP. For this study, the presence of IP was defined as CIPS ≥ 60 and we use an instrument validated in Spanish.^4^ We also use the Maslach Burnout Inventory (MBI) including three dimensions analysis (emotional exhaustion, depersonalization and personal achievement). The instrument assesses burnout prevalence in the target population through 22 items rated on a 7-point frequency scale. The questionnaire yields three numerical variables with the following cut-off points: Emotional Exhaustion (EE) is classified low (≤ 18), moderate (19‒26), or high (≥ 27); Depersonalization (DP) as low (≤ 5), moderate (6‒9), or high (≥ 10); and Personal Achievement (PA) as low (≤ 33), moderate (34‒39), or high (≥ 40). Burnout was identified when the following criteria were simultaneously met across the three dimensions of MBI; EE with a score ≥ 27, DP with a score ≥ 10, and PA with a score ≤ 33. We use an instrument validated in Spanish.^5^

We conducted descriptive statistical analyses of the variables included in the information questionnaire. We calculated the Pearson correlation coefficient to evaluate the association between the scores of the CIPS and the three dimensions of the MBI. We applied a logistic regression model to assess the relationship between the variables and the scores of the CIPS and the three dimensions of the MBI. All analyses were performed using Microsoft Excel® and GNU Operating System PSPP® version 2.0.1 for Windows®. A p-value < 0.05 was considered significant for hypothesis testing.

The age distribution of medical residents ranged from 25 to 40 years (mean = 30, SD = 2.86-years), including 38 females (68%) and 18 males (32%). Regarding the academic program, 39 residents (70%) were enrolled in Anesthesiology, while 17 residents (30%) were enrolled in Emergency Medicine. They reported duty time of 75.79 ± 23.78 hours per week and rest time of 26.8 ± 18.91 hours per week. Participants who scored in the frequent or intense IP range were considered to have IP, resulting in an overall IP prevalence of 45%. We observed an SB prevalence of 29% (16 participants with high EE and DP and low AP simultaneously) (Table 1).

**Table 1:** Frequency of Impostor Phenomenon and Burnout Syndrome and Pearson Correlation Coefficient of the participants.

Variable		Categories			Number of participants Total (n = 56)	
CIPS score		Few			9 (16%)	
		Moderate			22 (39%)	
		Frequently			18 (32%)	
		Intense			7 (13%)	
**IP Percentage Variable**					**Total (n = 25)**	
Gender		Male			7 (28%)	
		Female			18 (72%)	
Academic program		Anesthesiology			16 (64%)	
		Emergency Medicine			9 (36%)	
Academic year		Second			13 (52%)	
		Third			12 (48%)	
**MBI Score**					**Total (n = 56)**	
Emotional Exhaustion		Low (≤ 18)			10 (18%)	
		Moderate (19‒26)			13 (23%)	
		High (≥ 27)			33 (59%)	
Despersonalization		Low (≤ 5)			15 (27%)	
		Moderate (6‒9)			13 (23%)	
		High (≥ 10)			28 (50%)	
Personal achievement		Low (≤ 33)			21 (38%)	
		Moderate (34‒39)			26 (46%)	
		High (≥ 40)			9 (16%)	
**BS Percentage Variable**					**Total (n = 16)**	
Gender		Male			8 (50%)	
		Female			8 (50%)	
Academic program		Anesthesiology			10 (62%)	
		Emergency Medicine			6 (38%)	
Academic year		Second			8 (50%)	
		Third			8 (50%)	

Pearson correlation matrix of variables of interest (values refer to correlation coefficients *r*)						
	**EE MBI**		**DP MBI**	**PA MBI**		**CIPS**

EE MBI	1		‒	‒		‒
DP MBI	0.646167^a^		1	‒		‒
PA MBI	-0.509819^a^		-0.578983274^a^	1		‒
CIPS	0.4825796^a^		0.536297465^a^	-0.3530322^a^		1

a p < 0.05. BS, Burnout Syndrome; CIPS, Clance Impostor Phenomenon Scale; DP, Depersonalization; EE, Emotional Exhaustion; IP, Impostor Phenomenon; MBI, Maslach Burnout Inventory; PA, Personal Achievement.

In the analysis of association between IP and BS, a moderate non-causal correlation was obtained between EE and IP (95% CI 0.39‒1.15; *F* = 16.67); a strong non-causal correlation between DP and IP (95% CI 0.98‒2.46; *F* = 21.8); and a strong non-causal negative correlation between PA and IP (95% CI -1.75 to -0.28; *F* = 7.69) (p < 0.05). We obtained a strong non-causal correlation between the three dimensions of BS and IP (95% CI -0.88 to -2.255; *F* = 8.14) (Table 1), confirming the association between IP and the three dimensions of the BS which, to the best of our knowledge, has not been reported in this form in previous studies. There is a statistically significant correlation between the female gender and IP, as well as the depersonalization dimension of the MBI for BS. Additionally, a statistically significant correlation between the academic level and IP, as well the personal achievement dimension of the MBI for BS.

We identified the intense and frequent levels of IP among participants in this sample. When combined with a stressful and high-demand work environment, these factors necessitate the use of coping mechanisms such as perfectionism and overexertion, ultimately leading to BS. We report a higher prevalence of IP in the early stages of an academic career, associating a greater risk of IP with fewer years of practice. This suggests that increased work experience and the attainment of an academic degree, by reinforcing perceived competence, may help mitigate IP symptoms as part of a broader coping strategy.^6^

Among the three BS dimensions, PA is the one that best explains the BS score in our sample. Our population exhibited higher personal accomplishment scores, which served as a protective factor in reducing the frequency of BS diagnoses. PA can function as a coping mechanism that lowers the overall MBI score and, consequently, the BS diagnosis. It may also mask high exhaustion and depersonalization scores within the studied population.

Our findings suggest that the IP and BS are two interrelated mental health conditions that significantly affect medical residents enrolled in postgraduate academic programs. The statistical association observed between both constructs underscores the need for integrated approaches to their identification and management.

Given their strong link to work-related factors, we included variables such as the number of hours residents spent in hospital-based training activities. The reported weekly workload was consistent with findings from previous studies. However, we were unable to compare the self-reported rest hours with existing literature, likely due to the lack of standardized or validated instruments. Despite this limitation, our data highlight the importance of rest as a contributor to residents' overall quality of life.

This study was limited by its single-center design and inclusion of only two medical specialties, which restricts generalizability. Future studies should expand the range of specialties evaluated and incorporate validated tools for time-use assessment. Moreover, future research should explore the influence of personal, occupational, and motivational factors on the development of IP and BS, which may exert a greater effect than demographic characteristics alone. A deeper understanding of these variables could inform the development of targeted interventions to reduce the prevalence and impact of these conditions among medical trainees.

## Authors’ contribution

Cristina González Ramírez: Conceived and planned the study, carried out the observational study; designed the experimental study; processed the experimental data, performed the analysis, wrote and drafted the manuscript and designed the figures.

Rocío Pérez Bocanegra: Supervised the project; contributed to the interpretation of the results; provided critical feedback and helped shape the research, analysis and manuscript.

Carlos Armando Sánchez-Navarro: Responsible for all communications with the journal; conceived and planned the study, carried out the observational study; supervised the project; supervised the experimental data, performed the analysis; provided critical feedback and helped shape the research, analysis and manuscript.

All authors discussed the results and contributed to the final manuscript.

## Conflicts of interest

The authors declare no conflicts of interest.

## References

[1] Chrisman S.M., Pieper W.A., Clance P.R., Holland C.L., Glickauf-Hughes C. (1995). Validation of the Clance Imposter Phenomenon Scale. J Pers Assess.

[2] Maslach C., Leiter M.P. (2016). Understanding the Burnout experience: Recent Research and Its Implications for Psychiatry. World Psychiatry.

[3] Gottlieb M., Chung A., Battaglioli N., Sebok-Syer S.S., Kalantari A. (2019). Impostor syndrome among physicians and physicians in training: A scoping review. Med Educ.

[4] Sandoval-Lentisco A., Tortajada M., Palmero L.B., Campoy G., Fuentes L.J. (2024). Psychometric Properties of the Spanish Clance Impostor Scale (S-CIPS). Ann Psychol.

[5] Diaz E., Bon T. (2023). Validez y Fiabilidad del Inventario de Burnout de Maslach en México. Revista del Centro de Investigación de la Universidad la Salle.

[6] Lorello G.R., Schrewe B. (2024). Unmasking imposter syndrome: individual responsibility or repercussions of systemic oppression?. Br J Anaesth.

